# TIF1 Proteins in Genome Stability and Cancer

**DOI:** 10.3390/cancers12082094

**Published:** 2020-07-28

**Authors:** Roisin M. McAvera, Lisa J. Crawford

**Affiliations:** Patrick G Johnston Centre for Cancer Research, Queen’s University Belfast, Belfast BT9 7AE, UK; rmcavera01@qub.ac.uk

**Keywords:** TRIM24, TRIM28, TRIM33, TRIM66, genome stability, DNA damage, cancer

## Abstract

Genomic instability is a hallmark of cancer cells which results in excessive DNA damage. To counteract this, cells have evolved a tightly regulated DNA damage response (DDR) to rapidly sense DNA damage and promote its repair whilst halting cell cycle progression. The DDR functions predominantly within the context of chromatin and requires the action of chromatin-binding proteins to coordinate the appropriate response. TRIM24, TRIM28, TRIM33 and TRIM66 make up the transcriptional intermediary factor 1 (TIF1) family of chromatin-binding proteins, a subfamily of the large tripartite motif (TRIM) family of E3 ligases. All four TIF1 proteins are aberrantly expressed across numerous cancer types, and increasing evidence suggests that TIF1 family members can function to maintain genome stability by mediating chromatin-based responses to DNA damage. This review provides an overview of the TIF1 family in cancer, focusing on their roles in DNA repair, chromatin regulation and cell cycle regulation.

## 1. Introduction

Cells experience tens of thousands of insults to their DNA daily, through both endogenous and exogenous sources. If DNA damage is not properly dealt with, genome instability will arise, making cells susceptible to malignant transformation [1]. As a result, cells have evolved a tightly regulated DNA damage response (DDR)—a complex signalling cascade which aims to rapidly repair DNA whilst halting cell cycle progression. Alternatively, if the damage is deemed irreparable, the DDR will promote apoptosis [1]. Detection and repair of DNA damage occurs within the context of chromatin, the structure and function of which is regulated by post-translational modifications of histones. Research efforts over the past few decades have aimed to gain a better understanding of the proteins involved in its regulation.

TRIM24 (TIF1α), TRIM28 (TIF1β/KAP1), TRIM33 (TIF1γ) and TRIM66 (TIF1δ) comprise the transcriptional intermediary family 1 (TIF1) family of chromatin-binding proteins, a subfamily of the large, highly conserved tripartite-motif (TRIM) family of E3 ligases. All four TIF1 proteins have been shown to be aberrantly expressed or mutated in multiple cancer types; however, their role in cancer is still not fully understood. TIF1 proteins have many diverse functions, including a role in transcription, immunity, cell differentiation, DNA repair and mitosis, all of which can be altered in tumorigenesis [2,3,4,5,6,7]. Increasing evidence has indicated that TIF1 proteins contribute to the maintenance of genome stability. Here, we review their role in genome stability focusing on their alterations in cancer, role in DNA damage repair, cell cycle regulation and their interaction with histones.

## 2. TIF1 Protein Family Overview

TRIM proteins are a large family of over 80 proteins that are generally characterised by a conserved N-terminal RBBC motif made up of a RING domain, either one or two B-boxes (B1 and B2), a coiled-coil (CC) domain; and a highly variable C-terminal [8]. The RING domain confers E3 ligase activity, which can ubiquitinate substrates as part of the ubiquitin proteasome system (UPS) [9], whereas the B-box and CC domains mediate protein–protein interactions. TRIM proteins are further classified by their variable C-terminal domain into eleven subfamilies (C-I–C-XI) [10].

**Figure 1:**
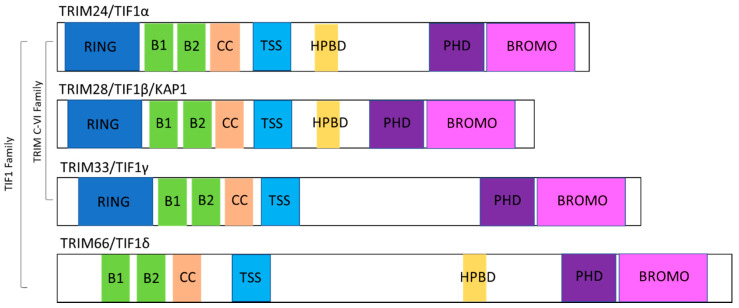
Schematic of the structure of TIF1 family proteins. RING—really interesting new gene, has intrinsic E3 ligase activity; B1 and B2—B-box domains; CC—coiled-coil domain; TSS—TIF1 signature sequence; HPBD—heterochromatin protein family binding domain; PHD—plant homeodomain finger; BROMO—bromodomain. TRIM24, TRIM28 and TRIM33 are classed as the C-VI subfamily of TRIM proteins based on their RBBC domain and PHD-bromodomain.

TRIM24, TRIM28 and TRIM33 are classified as the C-VI subfamily based on the presence of a plant-homeodomain (PHD) and bromodomain at the C-terminus. Together with TRIM66, which lacks a RING domain but similarly contains two B-boxes, a CC domain and a PHD-bromodomain, TRIM24, TRIM28 and TRIM33 can be alternatively classified as the TIF1 family of proteins. Through their PHD-bromodomain, TIF1 proteins can directly interact with modified histones; PHD fingers read the N-terminal domain of histone H3, normally the methylation status, whereas bromodomains read acetylated lysines [11,12]. In addition to their common PHD-bromodomain, all four TIF1 proteins contain a TIF-specific sequence (TSS) which is thought to be essential for transcriptional repression. Additionally, TRIM24, TRIM28 and TRIM66 have HP1 (heterochromatin protein 1) binding domains (HPBD) which allow the interaction of HP1 family proteins through a PxVxL motif. The structure of individual TIF1 proteins is depicted in Figure 1.

## 3. TIF1 Family Alterations in Cancer

Members of the TIF1 family are often dysregulated in human cancers, most commonly through altered gene expression, but also through deletions, translocations or loss-of-function mutations. TIF1 proteins exhibit both oncogenic and tumour-suppressive roles depending on the context, suggesting that their functions are cell-type specific. Given their altered expression in cancer, the TIF1 proteins have been proposed as predictors of prognosis and/or therapeutic targets, dependent on cell type and function. The alterations of each TIF1 protein in cancer is outlined below and summarised in Table 1.

### 3.1. TRIM24

Overexpression of TRIM24 is predominantly associated with cancer progression, inferring an oncogenic function. High expression of TRIM24 has been associated with a higher tumour grade in prostate cancer and was proposed to be an independent biomarker for prognosis [21]. Similarly, overexpression was observed in head and neck squamous cell carcinoma, glioblastoma, nasopharyngeal carcinoma and human cervical cancer, with high expression being associated with a more aggressive phenotype and tumour progression [13,14,15,20,47]. TRIM24 has also been reported as an oncogenic translocation partner of fibroblast growth factor receptor 1 (FGFR1) in 8p11 myeloproliferative syndrome [19]. Contrastingly, TRIM24 was reported to act as a tumour suppressor in murine hepatocellular carcinoma (HCC) by dysregulating retinoic acid receptor (RAR) signalling, and it was later shown that TRIM24, TRIM28 and TRIM33 can form regulatory complexes to suppress murine HCC [16,17]. 

### 3.2. TRIM28

Overexpression of TRIM28 has been observed in a number of cancers, whereby TRIM28 expression is higher in tumour tissue when compared to adjacent healthy tissue [7]. Like TRIM24, TRIM28 is typically considered as an oncogene. TRIM28 is overexpressed in glioma and was associated with poorer overall survival of patients [25]. Moreover, TRIM28 expression was high in ovarian cancer and correlated with aggressive clinical features [28]. Similar patterns have also been observed in breast and gastric cancer [22,24,48]. Conversely, loss-of-function TRIM28 mutations can predispose children to Wilms’ tumour, the most common type of renal cancer in paediatrics, suggesting a tumour suppressor role in this context [30]. Studies have been conflicting for the role of TRIM28 in the liver. Gene and protein levels of TRIM28 were higher in HCC tumours than their healthy counterparts, with high expression associated with a worse outcome for patients [26]. However, in a murine model, TRIM28 was reported to be a tumour suppressor in HCC [17]. In addition, Chen et al. [27] reported that in the early stages of lung cancer, TRIM28 expression was associated with better overall survival and reduced proliferation in vitro.

### 3.3. TRIM33

In contrast to the other TIF1 family members, TRIM33 has predominantly been identified as a tumour suppressor. As mentioned above, TRIM33 acts in conjunction with TRIM24 and TRIM28 to inhibit murine HCC [17]. Additionally, Pommier et al. reported that low TRIM33 expression is associated with increased genomic instability and subsequent cancer progression across multiple cancer types, including breast, pancreatic adenocarcinomas and clear cell renal cell carcinoma [33]. TRIM33 has been proposed as a prognostic predictor in breast cancer, where TRIM33 levels were reduced in both breast tissue and circulating plasma of breast cancer patients compared to healthy controls, and reduced TRIM33 expression was associated with worse overall survival [32]. However, in another study, patients with high TRIM33 showed poorer progression-free survival, indicating that there may be a diverse role for TRIM33 in breast cancer [31]. Moreover, TRIM33 also functions as a tumour suppressor in the haematological malignancies multiple myeloma (MM) and chronic myelomonocytic leukaemia (CMML) [35,38]. TRIM33 downregulation was identified as part of a 70-gene signature associated with high-risk MM [39], whereas in CMML DNA hypermethylation of the TRIM33 promoter region was observed in 35% of patients, leading to decreased expression [35]. Contrastingly, in B lymphoblastic leukaemia (B-ALL) TRIM33 plays an oncogenic role by preventing apoptosis [34] and TRIM33 expression was higher in advanced stages of colorectal cancer compared to earlier stages [36]. TRIM33 has also been proposed as a predictor of response to bromodomain and extra-terminal (BET) inhibitors in colorectal cancer [49]. BET inhibitors are a class of small molecule inhibitors that can reversibly bind BET family bromodomains to suppress transcription. In this study, loss of TRIM33 correlated with resistance to BET inhibitors in colorectal cancer [49].

### 3.4. TRIM66

An oncogenic role for TRIM66 in a number of cancers has been reported. TRIM66 expression was higher in osteosarcoma tissues than normal healthy tissue and was associated with poorer survival [45]. TRIM66 was also highly expressed in non-small cell lung carcinoma (NSCLC) and its expression correlated with metastasis [44]. Furthermore, in vitro studies have further shown that knockdown of TRIM66 reduces the proliferation of NSCLC, colorectal, HCC and prostate cancers, indicating an oncogenic role [42,43,46,50].

## 4. TIF1 Proteins and DNA Damage Repair

Cells sustain DNA damage through exposure to exogenous agents such as ultraviolet (UV), ionising radiation and genotoxic chemicals, and through endogenous processes such as metabolic activities and errors in DNA replication [1]. The DDR is a tightly regulated network of cellular pathways that functions to sense and repair the variety of DNA lesions that occur. Single-strand breaks (SSBs) are common and generally tolerated and repaired efficiently. However, double-strand breaks (DSBs), although less common, are much more toxic, as they can enable genomic rearrangement and are more complex to repair [51]. Hence, cells rely heavily on the DDR to prevent such genetic events. In healthy cells, DSBs are rapidly detected by the MRE11-RAD50-NBS1 (MRN) complex and the Ku70/Ku80 (Ku) heterodimer, activating a large signalling cascade [52]. Once a DSB has been detected, DDR signalling proteins are activated. The DDR is largely driven by phosphorylation events mediated by proteins from the phosphatidylinositol 3 kinase-like protein kinases (PIKKs) family—ATM, ATR and DNA-PKcs [53]. The MRN complex recruits ATM to the DSB, whereas the MRN complex recruits ATR and DNA-PKcs.

There are two major DNA repair pathways for DSBs—homologous recombination (HR) and non-homologous end joining (NHEJ). ATM kinase promotes HR repair, whereas ATR kinase promotes NHEJ, and DNA-PKcs is also essential for NHEJ. Since NHEJ utilises non-homologous regions of DNA to mediate DSB repair, it can act in any cell cycle phase but is prone to errors, often resulting in mutations. In contrast, HR utilises the sister chromatid as a template for repair in the S and G2 phases of the cell cycle and thus is less likely to result in error [51]. ATM has hundreds of other substrates, including histone variant H2AX at serine 139 (γH2AX), Chk2, topoisomerase II binding protein 1 (TopB1), breast cancer 1 early onset (BRCA1), TRIM24 and TRIM28 [53,54,55]. ATM phosphorylation of Chk2 activates the G1/S checkpoint, and, in addition, MDM2 on serine 395, stabilising p53. In response to replication stress, RPA (replication protein A) is recruited to single-stranded DNA, and ATR is consequently recruited and bound. ATR phosphorylates and activates Chk1, which in turn phosphorylates Cdc25a and promotes G2/M and intra-S checkpoints [53].

### 4.1. Localisation of TIF1 Proteins to Sites of DNA Damage

An early event in the DDR is the phosphorylation of H2AX by ATM. MDC1 (mediator of DNA damage checkpoint 1) can bind γH2AX, recruiting DDR proteins such as 53BP1 to the site of damage. Gong et al. [56] performed a screen of 32 human bromodomain-containing proteins in which TRIM24, TRIM28 and TRIM33 were all shown to localise to sites of damage in the osteosarcoma cell line U2OS after laser microirradiation. Further studies revealed that depletion of each of the four TIF1 proteins reduced the efficiency of HR repair with little impact on NHEJ, increased basal levels of γH2AX and increased the frequency of micronuclei, suggesting that they play a key role in orchestrating the DDR [57]. However, numerous studies have reported an increase in cells in the G1 phase upon TRIM24, TRIM28 or TRM66 knockdown, which may partly explain the reduction in HR since it cannot function in this phase [18,25,43,45]. Conversely, TRIM28 upregulation has also been shown to reduce HR efficiency, whilst promoting repair by NHEJ [58]. In this study, TRIM28 deacetylation enhanced TRIM28 interaction with 53BP1, thus promoting NHEJ. However, this function was independent of TRIM28 phosphorylation, indicating that the role of TRIM28 in the DDR is both ATM-dependent and ATM-independent. Although TRIM24, TRIM28 and TRIM33 can bind to form a complex to suppress HCC [17], there is currently no evidence that the three proteins work together during the DDR. TRIM24 and TRIM33 bind to each other in breast cancer cells, but this interaction remains unchanged in response to the DNA intercalating agent adriamycin or 10 Gy irradiation [54].

PARP1 and PARP2 can detect both SSBs and DSBs to catalyse the addition of poly (ADP-ribose) (PAR) chains on proteins localised to DNA damage sites or on to PARP itself (autoparylation). This induces structural changes in chromatin required for efficient DNA repair. TRIM33 is rapidly recruited to sites of DNA damage, where it interacts with the PAR-binding protein ALC1 (amplified in liver cancer-1) [59]. TRIM33 is required for the timely dissociation of ALC1 from chromatin and is recruited to damaged sites in a PARP1- and ALC1-dependent manner. TRIM33 recruitment was not dependent on ATM, ATR or DNA-PKcs but was dependent on an intact PHD-bromodomain [59]. Furthermore, shRNA knockdown of TRIM33 in HeLa cells lead to increased basal γH2AX, phosphorylated Chk2 and heightened sensitivity to bleomycin, a chemotherapeutic agent that induces single and double strand DNA breaks [59]. Similarly, we have shown that multiple myeloma cell lines with low TRIM33 expression have higher endogenous DNA damage as assessed by γH2AX levels [60]. TRIM33 is also required for the recruitment of CBX8 to sites of damage, another PARP-dependent DDR protein involved in both HR and NHEJ [61]. Contrastingly, in colorectal cancer cells knockdown of TRIM33 did not affect γH2AX levels, highlighting the cell-type specificity of TIF1 protein function [49].

Although TRIM66 was not reported to localise to sites of DNA damage following laser microirradation [56], siRNA knockdown of TRIM66 reduced the efficiency of HR repair in U2OS cells, although to a lesser extent than that of TRIM24, TRIM28 and TRIM33 [57]. In embryonic stem cells (ESCs) both shRNA knockdown and CRISPR/Cas9-mediated knockout of TRIM66 resulted in elevated basal DNA damage and genome instability as determined by γH2AX and Rad51 foci, comet assays and chromosome metaphase spreads [62]. Furthermore this depletion of TRIM66 sensitised ESCs to DNA damaging agents [62].

### 4.2. Phosphorylation of TRIM24 and TRIM28

**Figure 2:**
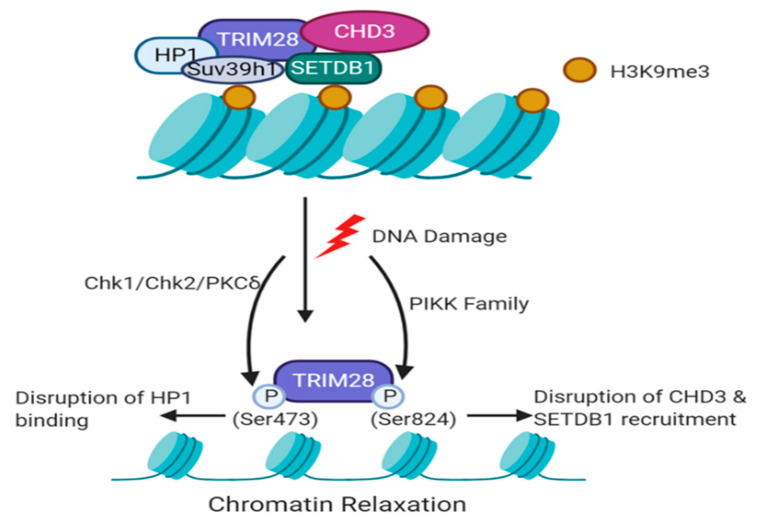
TRIM28 phosphorylation at double-strand breaks (DSBs)**.** TRIM28 forms a repressive complex with HP1 and methyltransferase Suv39h1 at heterochromatic H3K9me3 marks. In addition, TRIM28 also associates with the chromatin remodeller CHD3 and SETDB1 methyltransferase. Upon DNA damage, TRIM28 is phosphorylated at Ser473 by Chk1, Chk2 or PKCδ kinases, or at Ser824 by members of the PIKK family. TRIM28-Ser473 phosphorylation disrupts its binding to HP1 family proteins, inhibiting its transcriptional repressive activities. TRIM28-Ser824 phosphorylation disrupts the recruitment of CHD3 and SETDB1, promoting chromatin relaxation to facilitate the access of DNA repair factors.

Phosphorylation of TRIM28 at sites of DNA damage is an early event in the DDR and is mainly associated with the repair of DSBs in heterochromatin (Figure 2) [63,64,65,66]. White et al. described phosphorylation of TRIM28 at Ser824 in U2OS cells within 5 min of 9 Gy irradiation, which was diminished within 4 h [55]. This study proposed that ATM, ATR and DNA-PKcs could mediate Ser824 phosphorylation. However, multiple groups have identified TRIM28-Ser824 to mainly be a substrate of ATM [64]. TRIM28-Ser824 phosphorylation co-localised with the DDR proteins 53BP1, γH2AX, BRCA1 and TopBP1, and 53BP1 is required for formation of phosphorylated TRIM28 foci [55,66]. Phosphoproteomics analysis of protein phosphatase PP4 revealed that gene silencing of PP4 lead to increased phosphorylation of TRIM28 on Ser824 after 10 Gy of irradiation, which was independent of ATM activity [67]. This study proposed that the PP4C–R3b complex can dephosphorylate TRIM28 to regulate heterochromatic DNA repair to allow heterochromatin to return to its ‘normal’ state following DNA repair [67]. In melanoma, MAGE-C2 binds to TRIM28 to facilitate TRIM28-Ser824 phosphorylation and sufficient DNA repair in an ATM-dependent manner [68]. 

In addition, TRIM28 is also phosphorylated at Ser473 in response to DNA damage, which is required for efficient DNA repair [69]. Blasius et al. [70] first identified TRIM28-Ser473 as a DNA damage-induced substrate of Chk1 and Hu et al. [69] reported that Chk1 is indeed responsible for the phosphorylation of TRIM28-Ser473 in response to UV radiation. Alternatively, in response to etoposide, which immobilises topoisomerase II on DNA to induce damage, and IR-induced damage, Chk2 is the main kinase that mediates TRIM28-Ser473 phosphorylation [69]. Furthermore, protein kinase C-delta (PKCδ) can also phosphorylate TRIM28 at Ser473 [71]. Although phosphorylation of both TRIM28-Ser824 and Ser473 are observed during the DDR, their functions are independent of each other [63]. Phosphorylation of Ser824 precedes that of Ser473, and TRIM28-Ser473 phosphorylation has a much more diffuse localisation in the nucleus compared to Ser824, which localises to DNA damage foci [55,63]. Furthermore, DNA damage-induced phosphorylation of TRIM28-Ser473 attenuates TRIM28 binding to HP1 family proteins, proposing that TRIM28 binding to E2F1 increases and E2F1 activation of apoptotic proteins decreases [69]. The recruitment of TRIM28 to DSBs and phosphorylation of TRIM28 has also been shown to be PARP-dependent [72]. 

Similar to TRIM28, TRIM24 is also phosphorylated during the DDR [54]. TRIM24 is a negative regulator of the tetrameric tumour suppressor p53 which possesses transcriptional activities and is upregulated in response to cellular stress such as DNA damage. Upon DNA damage, ATM phosphorylates TRIM24 at Ser768, promoting autoubiquitination of TRIM24 and its subsequent degradation in the MCF7 breast cancer cell line [54]. ATM also phosphorylates p53, which can induce the transcription of TRIM24 to ultimately target phosphorylated p53 for degradation, terminating the DDR [54].

## 5. TIF1 Proteins, Histone Modifications and Chromatin Remodelling

To maintain its integrity, eukaryotic DNA is tightly packed into a state known as chromatin, a structure which limits accessibility of DNA to its binding partners. Central to chromatin is the nucleosome, which is composed of an octamer of the four core histones (H3, H4, H2A and H2B) around which DNA is wrapped [73]. Histones undergo post-translational modifications which can regulate chromatin dynamics and underlying DNA activity. Such histone modifications are fundamental to the maintenance of genome stability, and include phosphorylation, ubiquitination, SUMOylation, acetylation and methylation. As mentioned previously, phosphorylation of H2AX is an early event in the DDR. Generally, at DSB sites nucleosomes can obstruct the access of DNA repair factors and therefore chromatin structure must be altered for efficient repair [73]. Therefore, chromatin remodelling complexes such as CHD3 are recruited to relax chromatin and enhance their accessibility. 

Due to their PHD-bromodomain, TIF1 proteins can function as ‘readers’ of histones. Generally, bromodomains can recognise acetylated lysine residues of H3 and H4, whereas PHD fingers can read methylated lysine marks, particularly of H3K4 [11,12]. It is therefore not surprising that TIF1 proteins can mediate histone modifications to promote genome stability.

### 5.1. TRIM28-Dependent Recruitment of Histone Modifiers during the DDR

Many proteins that are involved in chromatin remodelling or histone modification have been identified to interact with TRIM28, including HP1, CHD3, HDAC1 and SETDB1 [6,74,75]. DSBs promote methylation of H3K9, and H3K9me3 is concentrated within heterochromatin, which is associated with gene repression. Loss of H3K9me2/me3 leads to failure to activate DNA repair, resulting in genomic instability. Ayrapetov et al. [72] have shown that TRIM28, HP1 proteins and the methyltransferase Suv39h1 form a repressive complex which is transiently recruited to damaged euchromatin, increasing H3K9me3 levels. This is vital for the acetyltransferase activity of Tip60 on ATM and thus activation of ATM. Furthermore, TRIM28 is known to mediate gene silencing through its interaction with the methyltransferase SETDB1, which specifically tri-methylates H3K9 [75]. Upon ATM activation, TRIM28 is phosphorylated and released from the TRIM28/HP1/Suv39h1 complex, allowing chromatin relaxation and access of DNA repair machinery such as 53BP1 and BRCA1 (Figure 2) [7,72]. Furthermore, ablation of the phosphorylation site Ser824 on TRIM28 leads to loss of chromatin relaxation and the inability to repair DSBs [64]. Since TRIM28 facilitates chromatin relaxation and accessibility of DNA modifying proteins, increased endogenous damage upon TRIM28 knockdown could be due to errors in transcription or DNA replication. However, this mechanism has not yet been explored in the context of the DDR.

A study has proposed that SET, an oncogene and histone chaperone, can interact with TRIM28 independent of DNA damage and regulate its retention to chromatin [76]. Overexpression of SET leads to an impaired DDR, retention of both TRIM28 and HP1 in chromatin and increased H3K9me3 in U2OS cells. It is therefore probable that SET regulates the retention of TRIM28 at DNA breaks, enabling its timely dissociation and subsequently relaxing chromatin to allow the access of DNA repair factors [76]. However further investigation into this mechanism is required.

SUMOylation of TRIM28 is required for its repressive function, and TRIM28 can undergo autoSUMOylation [7,77]. CHD3, a nucleosome remodeller, interacts with the SUMO1 motif of TRIM28. Upon irradiation, TRIM28-Ser824 is phosphorylated and interrupts CHD3-SUMO1 interaction, resulting in the dispersion of CHD3 from TRIM28, promoting chromatin relaxation and heterochromatic DNA repair [74]. TRIM28 can also from a complex with the histone deacetylase HDAC1 to inhibit viral HIV-1 integration [78]. In U2OS cells and human fibroblasts, depletion of TRIM28 resulted in global hyperacetylation of H4 [79]. As HDAC1/2 are recruited to DNA lesions where they can regulate acetylation of histone marks such as H3K56, further investigation into TRIM28-HDAC1 interaction could reveal a role in genome stability [80].

### 5.2. Histone Modifications Mediated by TRIM66 at DSBs

**Figure 3:**
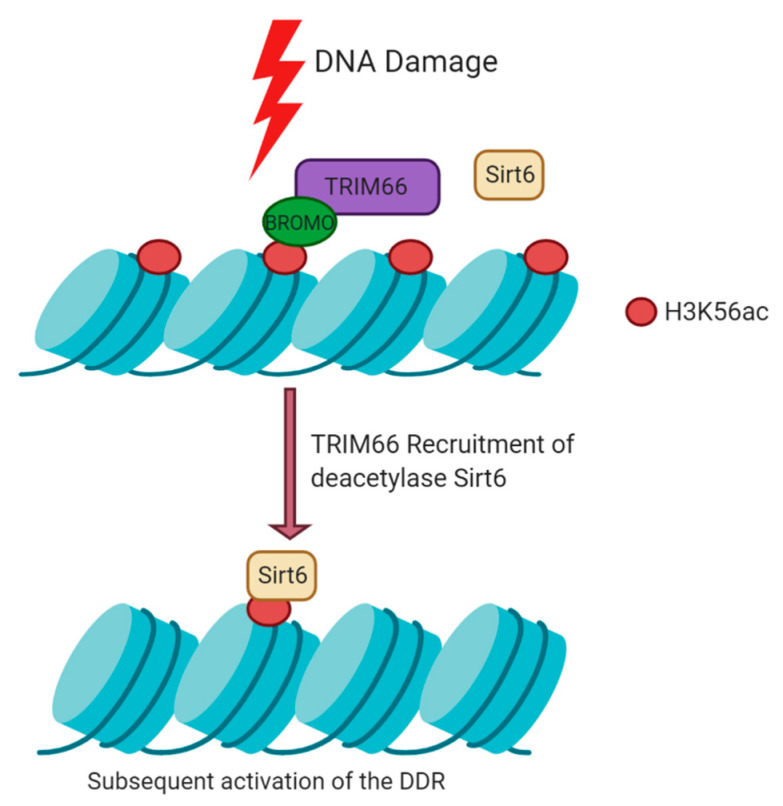
TRIM66 Response to DNA damage. In response to damage, TRIM66 is recruited to damage sites, where it recognises H3K56ac via its bromodomain (BROMO) and unmodified H3R2K4 via its PHD domain (not shown). TRIM66 recruits the deacetylase Sirt6 to rapidly remove H3K56ac marks to facilitate DNA repair. These marks are restored at later stages of DNA repair.

At DSBs, H3K56ac is rapidly deacetylated and subsequently restored at later stages of DNA repair. Loss of TRIM66 in ESCs leads to the retention of H3K56ac and elevated DNA damage levels [62]. The bromodomain of TRIM66 recognises H3K56ac at damaged sites, recruits the deacetylase Sirt6 to chromatin and thus activates the DDR (Figure 3) [62]. TRIM66 can also read unmodified H3R2K4 in response to damage via its PHD finger [62]. Together, this proposes the model that TRIM66 is a dual reader of H3K56ac and unmodified H3R2K4, upon damage, to recruit Sirt6 and ultimately maintain genome stability through histone deacetylation [62]. TRIM66 can also bind the HP1 isotype HP1γ, which is thought to contribute to its ability to promote condensed chromatin [81].

### 5.3. TRIM24 and TRIM33 as Histone Readers

Currently, it is unclear if TRIM24 and TRIM33 can read histone marks or recruit chromatin remodellers specifically in response to DNA lesions like their family members TRIM28 and TRIM66. TRIM24 can preferentially bind H3K4me0/H3K23ac marks on chromatin [82]. Its PHD domain recognises H3K4me0, while simultaneously its bromodomain binds H3K23ac. TRIM24 also interacts with HDAC1 to suppress HCC and interacts with other histone marks such as H3K9ac, H3K14ac, H3K27ac and H4ac in breast cancer [17,82]. Given the role of HDAC1 and histone modifications in response to DNA damage, it is possible that TRIM24 may read these marks as part of the DDR.

TRIM33 has been identified as a reader of the heterochromatic mark H3K9me3. This was discovered in the context of ESCs, where it was shown that TRIM33 can displace HP1 to bind H3K9me3 in order to allow mesodermal differentiation [83]. The bromodomain of TRIM33 can recognise H3K18ac, and thus TRIM33 can act as a dual reader of K9me3 and K18ac marks [83]. DSBs can promote the methylation of H3K9 to repress chromatin for DNA repair [72]. Given the reports that TRIM33 is recruited to DSBs [56,59], it may be possible that TRIM33 can read H3K9me3 at DSBs. In addition, Sirt7 can deacetylate H3K18ac to promote DNA repair [84]. It is possible that TRIM33 regulation of H3K18ac marks through Sirt7 may therefore also contribute to the maintenance of genome stability. However, the regulation of histone modifications by TIF1 members TRIM33 and TRIM24 during the DDR remains unknown.

## 6. TIF1 Proteins and Regulation of the Cell Cycle

The regulation of the cell cycle is key to maintaining genomic stability. The cell cycle is a highly organised process that ensures the precise replication of genetic material and subsequent cell division. The cell cycle has four distinct phases; G1, S, G2 and M [85]. G1, S and G2 comprise interphase, during which cells grow and duplicate their DNA in preparation for cellular division during the next phase of mitosis (the M-phase). Mitosis can be further subdivided into prophase, metaphase, anaphase and telophase. In brief, chromosomes become condensed and attach to the mitotic spindle, and sister chromatids are pulled to opposite poles of the cell to enable division into two identical daughter cells [86].

Progression through all of these phases is tightly regulated by cyclins, cyclin-dependent kinases (CDKs) and cell cycle checkpoints, which sense potential defects during DNA synthesis and chromosome segregation. Such defects result in cell cycle arrest until the defect is repaired, or if irreparable result in cell death. In response to DNA damage, Chk2 and p53 can arrest cells in G1 phase (G1/S checkpoint), or Chk1 can do so in S or G2 phase (Intra-S checkpoint and G2/M checkpoints, respectively) [85]. Additionally, the spindle assembly checkpoint (SAC) prevents defects in chromosome segregation during mitosis to prevent chromosomal instability [87]. Defects in any of these checkpoints, regulatory proteins or DNA repair machinery ultimately result in genomic instability.

### 6.1. G1 to S-Phase Transition 

A common effect observed when TIF1 proteins are silenced is a reduction of cells in the S or G2/M phase and an increase in G1 as knockdown of TRIM24, TRIM28 or TRIM66 results in cell cycle arrest [18,25,43,45]. However, knockdown of TRIM33 can increase the growth rate of cancer cells in line with its tumour suppressor function [33].

Not only is the phosphorylation of TRIM28 important for faithful DNA repair, it is also involved in the regulation of the cell cycle. Specifically, it is involved in the transition from G1 phase to S-phase with shRNA knockdown of TRIM28 leading to a G1 phase arrest in glioma independent of DNA damage [25,71]. Phosphorylation of TRIM28-Ser473 increases as undamaged cells transition from G1 phase to early S-phase, as does the gene expression of cyclin A2. TRIM28 binds the promoter of cyclin A2 when TRIM28-Ser473 remains non-phosphorylated, thus repressing cyclin A2 expression [71]. Additionally, phosphorylation of TRIM28-Ser473 disrupts TRIM28-HP1 interaction due to the close proximity of Ser473 to the HP1-binding domain of TRIM28. This subsequently upregulates the expression of Cdc2 and Cdc25A, allowing cell cycle progression [71]. Since TRIM28-Ser473 phosphorylation also increases during the DDR, investigation into its regulation of the cell cycle post-damage could reveal additional mechanisms. Perhaps cell progression to S-phase aims to promote error-free DNA repair by HR. 

### 6.2. Regulation of p53

The tetrameric transcriptional activator p53 is one of the most studied proteins in cancer due to its high mutation rate across most cancer types and its role as a tumour suppressor [88]. p53 is phosphorylated at Ser15 in response to DNA damage and can promote cell-cycle arrest—both at G1/S and G2/M, with apoptosis and DNA repair making it a crucial factor for genome stability [88,89]. The RING E3 ligase MDM2 is the major negative regulator of p53, as it can ubiquitinate and therefore target p53 for degradation [88,90]. MDM2 can also inhibit p53 acetylation [91]. TRIM28 does not directly bind p53, but rather interacts with MDM2 through its CC domain and is thus targeted to p53 in vivo [92]. This interaction promotes p53 ubiquitination and deacetylation, thus inhibiting p53 function. Additionally, TRIM28 knockdown lead to p53 activation [92]. In both breast and colon cancer cell lines TRIM28 knockdown enhanced p21 levels, a target gene of p53, following actinomycin D treatment or irradiation resulting in a reduction of cells entering S-phase [93].

Increased p21 levels were also observed basally in the absence of TRIM33 in HeLa cells [59] but were reduced in transformed NMuMG (normal murine mammary gland) cells [33]. In transformed NMuMG cells this was due to reduced p53 transcriptional activity and reduced active p53 (p53-Ser15 phosphorylation). In osteosarcoma, TRIM66 depletion upregulates p53 expression, but the mechanism is unclear [45]. TRIM24 has been identified as a binding partner of p53. Alton et al. [94] first revealed this interaction by mass spectrometry peptide analysis of tagged-p53 complexes in ESCs. TRIM24 acts as negative regulator of p53 by ubiquitinating and targeting it for degradation. TRIM24 depletion also induced p53-dependent apoptosis in breast cancer cells [94]. Later studies revealed that after DNA damage TRIM24 forms part of an autoregulatory feedback loop for p53 [54].

**Figure 4:**
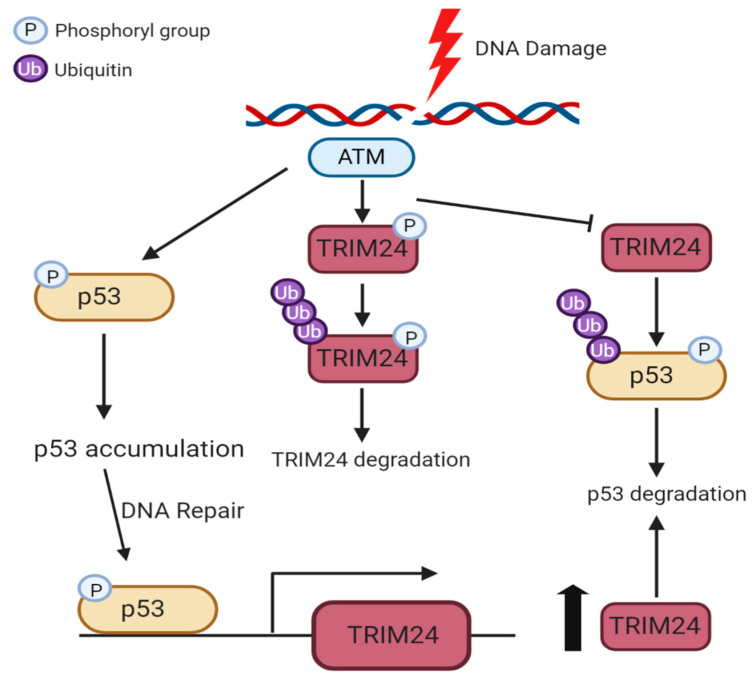
The TRIM24-p53 autoregulatory feedback loop. In response to DNA damage, the ATM kinase is activated, which phosphorylates both p53 and TRIM24. Phosphorylated TRIM24 becomes auto-ubiquitinated via its RING domain and is targeted for proteasomal degradation. p53 is not ubiquitinated by TRIM24 and becomes stabilised. Phosphorylated p53 accumulates and functions to promote DNA repair. Post-DNA damage, phosphorylated p53 can then bind the promoter of TRIM24, activating TRIM24 transcription and increasing its expression. Newly synthesised TRIM24 now selectively targets phosphorylated p53 for degradation in order to bring excess p53 levels back to a normal level, thus completing the negative feedback loop.

Phosphorylation of TRIM24 by ATM disrupts its interaction with p53 and ultimately results in TRIM24 degradation. This is later reversed when phosphorylated p53 upregulates TRIM24 expression and p53 is again targeted for degradation, forming the negative feedback loop (Figure 4) [54].

### 6.3. TRIM33 Regulation of Mitotic Checkpoints

Chromosome segregation and exit of mitosis are required for cell cycle progression. The anaphase promoting complex/cyclosome (APC/C) is an E3 ligase that, once phosphorylated, is activated and associates with the Cdc20 protein, promoting the segregation of sister chromatids and mitotic exit once chromatids are correctly attached to the spindle. This process is regulated by the spindle assembly checkpoint (SAC), which remains activated until microtubules are correctly attached to kinetochores, thereby inhibiting the APC/C [95]. Hence, metaphase-to-anaphase transition during mitosis requires inactivation of the SAC.

Sedgwick et al. [96] identified TRIM33 as an APC/C-interacting protein. In their study, TRIM33 was transiently depleted in HeLa cells using siRNA and these cells failed to undergo metaphase-to-anaphase transition. This was due to defective APC/C-Cdc20 activity and prolonged SAC activation [96]. Pommier et al. [33] built upon these findings, investigating prolonged shRNA knockdown of TRIM33 in immortalised mouse embryonic fibroblasts (MEFs) and transformed NMuMG cells. Indeed, transient inactivation of TRIM33 led to a mitotic blockade due to SAC activation, in agreement with Sedgwick et al. [96].

**Figure 5:**
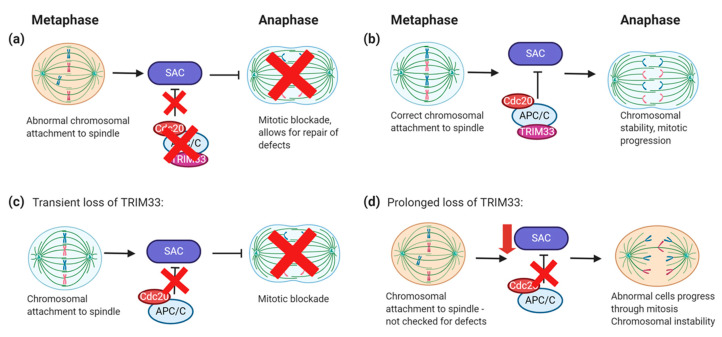
Regulation of the spindle assembly checkpoint (SAC) by TRIM33. During metaphase, chromosomes attach to microtubules and align along the equator. Sister chromatids must be attached to opposite spindle poles to ensure that when segregated, each daughter cell receives one copy of the chromosome. (**a**) Chromosomes are incorrectly attached to the spindle. The SAC remains active, whereas the APC/C/Cdc20/TRIM33 complex is inactive. This results in mitotic blockade, thus cells do not progress from metaphase to anaphase. (**b**) When the chromosomes are correctly attached, the APC/C/Cdc20/TRIM33 complex is activated and inactivates the SAC, allowing mitotic progression. (**c**) Transient loss of TRIM33 reduces APC/C/Cdc20 activity and thus enhanced SAC activation, resulting in a mitotic blockade. (**d**) Prolonged loss of TRIM33 results in eventual SAC inactivation, thus cells with incorrect chromosomal attachment can bypass the SAC, resulting in defective cells and increased chromosomal instability.

However, long-term TRIM33 inactivation resulted in attenuation of the SAC, which led to the accumulation of chromosomal abnormalities, including micronuclei, lagging chromosomes and multinucleated cells (Figure 5) [33]. In vivo studies further demonstrated this, with TRIM33 loss driving tumour progression [33]. Reduced activity of the p53-depedent postmitotic checkpoint also contributed to this observed genomic instability. Additionally, SAC and DDR components have been shown to work together to ensure genomic integrity [97]. It may therefore be possible that SAC inactivation in TRIM33-deficient cells may also contribute to the higher endogenous damage observed in other studies. Analysis of human cancer cell lines and patient data revealed that reduced TRIM33 expression correlates with increased genomic rearrangements, further indicating that TRIM33 regulates chromosomal stability [33]. In agreement with these findings, we have similarly demonstrated that MM patients with loss of TRIM33 display an increased frequency of genomic rearrangements [60].

## 7. Potential Therapeutic Exploitation of TIF1 Proteins in Cancer

Given their altered expression in cancers, TIF1 proteins have been proposed as both potential therapeutic targets and prognostic markers for cancer. It has been shown, for TRIM33 at least, that its bromodomain is required for recruitment to DNA lesions and this may be likely for its fellow family members [59]. Recently, small molecule inhibitors against the BET class of bromodomains have shown anti-cancer activities [98]. Shi et al. [49] reported that loss of TRIM33 or TRIM24 can lead to resistance to potent BET inhibitors (BETi) JQ1 and GS-626510 in colorectal cancer, suggesting both TRIM24 and TRIM33 can be directly targeted by BETi. This opens up the possibility of using readily available BETi in cancers where TIF1 proteins are overexpressed. Given that knockdown of all TIF1 proteins has sensitised cells to DNA damaging agents, BETi could be used to sensitise cancer cells to such agents. In addition, more specific inhibitors against TIF1 bromodomains may be an attractive target, and TRIM24 bromodomain inhibitors are in the early stages of development [99].

PARP inhibitors (PARPi) have proven successful in ovarian and breast cancers with mutated *BRCA1* or *BRCA2* genes [52]. Both TRIM28 and TRIM33 recruitment to DNA damage sites are dependent on PARP1. Thus, again, a combination of PARPi and other DNA damaging agents may be an option for patients with high expression of TIF1 proteins. Additionally, where reduced expression is observed in cancers, for example TRIM33, this could potentially be utilised as a biomarker for predicting prognosis or cancer stage, or to stratify patients who may respond to DNA damaging agents or BETi.

MDM2 RING domain inhibitors have been shown to stabilise and activate p53 by preventing p53-MDM2 binding, and in leukaemia cells this resulted in apoptosis [100,101]. Since TRIM28 can bind MDM2 via its CC domain to promote p53 binding [92], perhaps targeting this interaction could induce a similar effect. Additionally, the RING domain of TRIM24 could also be an inhibition target to stabilise p53 levels. However, given the large number of TRIM E3 ligases that exist, targeting these particular domains may be largely unspecific and difficult.

## 8. Conclusions and Future Perspective

TIF1 proteins play a complex role in cancer cells due to their diverse range of functions. In this review we have highlighted their key role in maintaining genome stability through their involvement in the DDR, chromatin regulation and cell cycle regulation. Depending on the cell-type TIF1 proteins may have dual roles as either oncogenes or tumour suppressors. Their ability to regulate p53 and cell cycle checkpoints, and to promote DNA repair through E3 ligase activity, post-translational modifications and interactions with chromatin, can all contribute to these dual roles in cancer.

TRIM24, TRIM28 and TRIM66 are most commonly overexpressed in cancer, raising the possibility of targeting them for anti-cancer therapy. Perhaps future research could lead to inhibition of TIF1 bromodomains as a target. Furthermore, knockdown of all four TIF1 proteins individually sensitises cancer cells to DNA damaging agents, raising the possibility of their inhibition in combination with such agents. Additionally, where reduced expression is observed in cancers, TIF1 proteins may be useful for predicting prognosis and therapeutic response for patients. Ultimately, a better understanding of the function of TIF1 proteins in genome stability could lead to novel cancer therapeutic targets and/or patient stratification.

## Figures and Tables

**Table 1:** Alterations in the TIF1 family members in cancer, and the proposed function of that gene on cancer progression. ^1^ WT—Wilms’ tumour; ^2^ B-ALL—B-cell acute lymphoblastic leukaemia; ^3^ CMML—chronic myelomonocytic leukaemia; ^4^ MM—multiple myeloma; ^5^ Fibroblast growth factor receptor 1.

TIF1 Member	Cancer Type	Alteration	Observed Function	Reference
*TRIM24*	Cervical	High expression	Oncogene	[13]
	Glioma	High expression	Oncogene	[14]
	Head & Neck	High expression	Oncogene	[15]
	Liver	Gene deletion	Tumour suppressor	[16]
	Liver	N/A	Tumour suppressor	[17]
	Lung	High expression	Oncogene	[18]
	Myeloproliferative syndrome	Chromosome translocation: FGFR1 ^5^	Oncogene	[19]
	Nasopharyngeal	High expression	Oncogene	[20]
	Prostate	High expression	Oncogene	[21]
*TRIM28*	Breast	High expression	Oncogene	[22,23]
	Gastric	High expression	Oncogene	[24]
	Glioma	High expression	Oncogene	[25]
	Liver	High expression	Oncogene	[26]
	Liver	N/A	Tumour suppressor	[17]
	Lung (early stage)	High expression	Tumour suppressor	[27]
	Ovarian	High expression	Oncogene	[28]
	Pancreatic	High expression	Oncogene	[29]
	Renal (WT ^1^)	Loss-of-function mutation	Tumour suppressor	[30]
*TRIM33*	Breast	High expression	Oncogene	[31]
	Breast	Reduced expression	Tumour suppressor	[32,33]
	B-ALL ^2^	N/A	Oncogene	[34]
	CMML ^3^	Reduced expression	Tumour suppressor	[35]
	Colorectal	High expression	Oncogene	[36]
	Liver	Reduced expression	Tumour suppressor	[37]
	MM ^4^	Reduced expression	Tumour suppressor	[38,39]
	Pancreatic	Reduced expression	Tumour suppressor	[33,40]
	Renal	Reduced expression	Tumour suppressor	[33,41]
*TRIM66*	Colorectal	High expression	Oncogene	[42]
	Liver	High expression	Oncogene	[43]
	Lung	High expression	Oncogene	[44]
	Osteosarcoma	High expression	Oncogene	[45]
	Prostate	N/A	Oncogene	[46]
